# Seroprevalence to Measles Virus after Vaccination or Natural Infection in an Adult Population, in Italy

**DOI:** 10.3390/vaccines8010066

**Published:** 2020-02-03

**Authors:** Gabriele Anichini, Claudia Gandolfo, Simonetta Fabrizi, Giovan Battista Miceli, Chiara Terrosi, Gianni Gori Savellini, Shibily Prathyumnan, Daniela Orsi, Giuseppe Battista, Maria Grazia Cusi

**Affiliations:** 1 Department of Medical Biotechnologies, University of Siena, Santa Maria delle Scotte Hospital, V.le Bracci, 1 53100 Siena, Italy; gabriele.anichini@student.unisi.it (G.A.); claudia.gandolfo@unisi.it (C.G.); chiara.terrosi@unisi.it (C.T.); gianni.gori@unisi.it (G.G.S.); shibilyps@gmail.com (S.P.); 2 Preventive Medicine and Health Surveillance Unit, Santa Maria delle Scotte Hospital, V.le Bracci, 1 53100 Siena, Italy; s.fabrizi@ao-siena.toscana.it (S.F.); giovanni.miceli@ao-siena.toscana.it (G.B.M.); daniela.orsi@unisi.it (D.O.); giuseppe.battista@unisi.it (G.B.)

**Keywords:** measles virus, vaccine, neutralizing antibodies, seroprevalence

## Abstract

An increase in measles cases worldwide, with outbreaks, has been registered in the last few years, despite the availability of a safe and highly efficacious vaccine. In addition to an inadequate vaccination coverage, even in high-income European countries studies proved that some vaccinated people were also found seronegative years after vaccination, thus increasing the number of people susceptible to measles infection. In this study, we evaluated the immunization status and the seroprevalence of measles antibodies among 1092 healthy adults, either vaccinated or naturally infected, in order to investigate the persistence of anti-measles IgG. Among subjects who received two doses of measles vaccine, the neutralizing antibody titer tended to decline over time. In addition, data collected from a neutralization assay performed on 110 healthy vaccinated subjects suggested an inverse correlation between neutralizing antibody titers and the time elapsed between the two vaccinations, with a significant decline in the neutralizing titer when the interval between the two doses was ≥11 years. On the basis of these results, monitoring the serological status of the population 10–12 years after vaccination could be important both to limit the number of people who are potentially susceptible to measles, despite the high efficacy of MMR vaccine, and to recommend a booster vaccine for the seronegatives.

## 1. Introduction

Measles virus (MV) is a negative single-stranded RNA virus belonging to the Morbillivirus genus, *Paramixoviridae* family [1]. It is the causative agent of a highly contagious acute infectious disease, typical of infancy, characterized by fever, skin rash, cough, coryza, conjunctivitis and a generalized immune suppression [1]. The virus is transmitted by large respiratory droplets, it spreads in the respiratory route and in regional lymph nodes, thus resulting in lymphatic and hematic dissemination with appearance of first clinical signs after 9–19 days [2]. Recovery is followed by lifelong immunity to measles. In rare cases, severe measles-associated central nervous system (CNS) complications may develop [3]. MV infection is also responsible for a transient immune suppression that may last longer than two years after infection and it leads to opportunistic infections [4] and to life-threatening complications, such as pneumonia and/or gastrointestinal disease [5,6]. Nevertheless, this disease is associated with the induction of a strong and specific life-long immune response to the virus [7]. There is no specific antiviral treatment against measles, thus the prophylactic vaccine is considered the best strategy to prevent this virus infection [8]. Furthermore, the monotypic nature of the virus and the lack of an animal reservoir make measles a considerable candidate for eradication [9]. In Italy, a single-antigen measles vaccine became commercially available in 1976 and its administration has been recommended by the Ministry of Health since 1979, with one dose for children aged 15 months. In the early 1990s, the trivalent measles-mumps-rubella (MMR) vaccine containing a live attenuated Edmonston B strain was recommended for administration at 12 months of age. Since 2003, the national vaccination schedule has recommended two doses of MMR vaccine in all Italian regions: The first at 12–15 months and the second at six years or older, only for those who had already received one dose and were older than six years at that date [10,11]. Subsequently, due to the lower MMR vaccination coverage (<90%) in Italy, especially among infants and adolescents [12], and the occurrence of a large measles outbreak in January 2017, a new law was passed and adopted in July 2017. This law extended the number of mandatory vaccines from four to ten, including MMR, administered at 13–15 months and six years [13]. Since then, the attenuated varicella strain has been included in the formulation of the vaccine. This can be administered at the same session as trivalent anti-measles-mumps-rubella plus the monovalent anti-varicella vaccine or as quadrivalent MMRV combined vaccine [14].

In spite of this, according to the latest update on measles circulation by ECDC, 29 EU/EEA Member States reported 13,331 cases of measles, from October 2018 to September 2019, 10,541 (79%) of which were laboratory-confirmed. No countries reported zero cases during the 12-month period. The highest number of cases were reported by France (2699), Italy (1845), Poland (1811), and Romania (1485), accounting for 20%, 14%, 12%, and 11% of all cases, respectively [15]. Measles outbreaks mostly occurred in unvaccinated individuals, thus a high vaccination coverage is the most important goal to prevent the disease. Epidemiologic studies have shown that the level of functional neutralizing antibodies at the time of exposure to the wild-type (WT) virus during a measles outbreak is a good correlate of protection from infection, with higher titers needed to prevent infection rather than to prevent the disease [16]. According to literature, levels of anti-measles antibodies tend to decline over the life course, as demonstrated by measuring the level of measles neutralizing antibodies in the subjects’ sera at different times after vaccination [17,18,19,20]. Moreover, this phenomenon appears to occur faster following vaccination rather than after naturally acquired infection [21,22]. Thus, it is important to better understand vaccine-induced antibody persistence and how persistence patterns may influence the risk of vaccine failure. In this study, we report data concerning the seroprevalence of a healthy population sample, analyzing 1092 sera among healthy adults, the immunogenicity of the vaccine and the protective antibody levels to measles virus after vaccination or natural exposure to the virus.

## 2. Materials and Methods

### 2.1. Study Population

**Figure 1:**
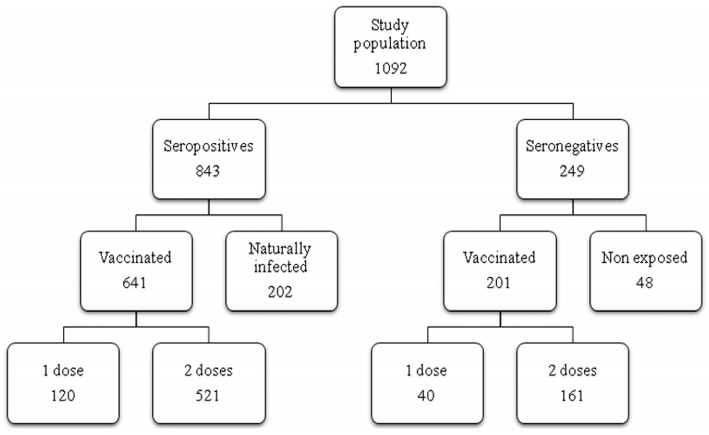
Flow diagram of the study population. Schematic representation of the study population enrolled in the study with the relative number of subjects in each group.

The participants in this observational study were students, postgraduates, medical doctorsand health care workers subjected to routine analysis for the biological risk assessment at the Center of Preventive Medicine and Health Surveillance of the University Hospital ‘Santa Maria alle Scotte’ in Siena between January 2018 and May 2019. Among the participants, some subjects presented their history of vaccination against measles, some had never been vaccinated and others had a history of measles infection. This research was carried out according to the principles of Helsinki declaration. Ethical approval was obtained from the local Ethical Committee for clinical trials (approval n° 11466_2017) (Comitato Etico Regione Toscana*-*Area Vasta Sud Est) in terms of General Data Protection and Regulation (GDPR) upon written informed consent signed by all subjects prior to participating in this study [23,24]. A total of 1092 subjects, 361 males and 731 females, (mean age 27.1 years; CI 95% 26.6–27.6) were screened for anti-measles IgG. All subjects born before 1977 declared to have contracted measles infection; the others, born later, were distinguished into three groups, according to their vaccination records: vaccinated with one or two doses and nonvaccinated (Figure 1). Lastly, among those born after 1977, 110 sera of subjects (mean age 24.9 years; CI 95% 24.1–25.7) vaccinated with two doses of measles vaccine, either monovalent (Moraten, Istituto sieroterapico e vaccinogeno svizzero, Berna, Switzerland) or trivalent (measles, mumps and rubella (MMR) Priorix (GlaxoSmithKline, Verona, Italy) were analyzed for the titer of specific neutralizing antibodies. These titers were compared with 100 samples of subjects (mean age 48.6 years; CI 95% 46.2–51.1), who had been exposed to natural infection. 

### 2.2. Cells and Viruses 

Vero cells (ATCC CCL-81) were grown as a monolayer in Dulbecco’s modified Eagle’s medium (DMEM) (Euroclone, Milan, Italy) supplemented with 100 U/mL penicillin/streptomycin (Euroclone) and 5% heat-inactivated fetal calf serum (FCS) (Euroclone) at 37 °C in a humidified 5% CO2 atmosphere. Measles virus Edmonston B strain (ATCC VR-24) was propagated on Vero cells until a cytopathic effect (CPE) appeared. Viral stocks were prepared, titrated on Vero cells, and stored at −80 °C for long term.

### 2.3. Measles IgM/IgG Antibody Detection

Sera obtained from subjects were analyzed for the presence of measles specific IgM/IgG antibodies to the recombinant MV nucleoprotein, by LIAISON XL (Liaison Measles IgG/IgM, DiaSorin, Saluggia, Italy), a chemiluminescence analyzer using paramagnetic solid phase microparticles. Threshold IgG values regarded as positive immune status were >16.5 AU (Arbitrary Unit)/mL, with detection limit of 13.5 AU/mL; while IgM threshold for the presence of measles infection was >1.1 AU/mL, with detection limit at 0.9 AU/mL.

### 2.4. Measles Microneutralization Test

The measles virus neutralization assay was carried out on Vero cells in a 96-well microplate. Twenty-five microliters of 2-fold serial dilutions (1:8 to 1:1024) of vaccinated or naturally infected people sera were added to an equal volume of the Edmonston B strain MV containing 250 TCID_50_ and incubated for 90 min at 37 °C. Finally, 50 μL of Vero cells suspension (2 × 10^5^ cells/mL) prepared in a complete DMEM (Euroclone) medium were added to each well. Five days after incubation at 37 °C, the cultures were microscopically examined for the presence of CPE. The 50% end point titer of the serum neutralizing titer was calculated using the Reed and Muench method [25]. Serum samples with neutralizing titers of less than eight were considered negative [26]. A positive and negative control serum (Liaison Measles IgG Ctr) were included in each assay.

### 2.5. Statistical Analysis

Seroprevalence was calculated as the ratio between the number of positive test results and the number of performed tests. Geometric mean titers (GMTs), obtained by the neutralization assay, were calculated as log-transformed reciprocal titers and reported as back-transformed for each subclass. Differences between vaccination status, sex, time elapsed between dose one and two of the vaccine and time elapsed since the second dose of vaccine, and the last serological measles investigation, were evaluated. Furthermore, statistical significances were assessed with the two-tailed chi-squared test. Results were considered statistically significant at *p* < 0.05. Spearman’s rank correlation coefficient was used to assess correlations of log-transformed continuous variables by the group.

## 3. Results

### 3.1. Study Group

We enrolled 1092 subjects, who were screened for specific anti-measles IgG. Out of 1092 subjects, 843 (77.2%) were seropositive and 249 (22.8%) seronegative to the measles virus (Figure 1). The mean age of vaccinated subjects was 24.9 years (CI 95% CI 24.1–25.7). Among the nonvaccinated subjects, seropositives (naturally infected) and seronegatives (never exposed to the virus) had, respectively, a mean age of 39.0 years (95% CI 37.1–40.9) and 26.1 years (95% CI 24.6–27.4) (*p* < 0.00001). Vaccination coverage with one or two doses of vaccine of this population sample was estimated 77.1% (842/1092), lower than the 90%–95% threshold required for achieving herd immunity. The enrolled people were lately divided according to their vaccination history (one or two doses) or nonvaccinated (naturally infected or nonexposed to the virus) (Figure 1). Surprisingly, among those who received two doses of vaccine (as recommended by the Italian Ministry of Health since 2003), 161 out of 682 subjects (23.6%) were seronegative after vaccination. Except for one subject, who had not responded to the trivalent vaccine, the others were seropositive to mumps and rubella viruses, indicating that these individuals had responded to the vaccine. Surprisingly, this percentage was similar to that observed for vaccines with only one dose (40/160, 25%) (Figure 1). No significant differences in the seroprevalence rates were found in all the groups with respect to gender (*p* > 0.05).

### 3.2. Age-Specific IgG prevalence

**Figure 2:**
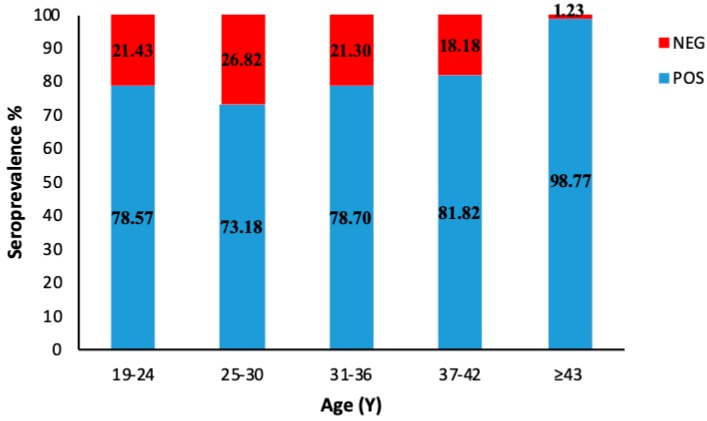
Age-specific measles IgG prevalence categorized into different age groups. Subjects over 43 years old were included into the same group.

In order to analyze the IgG prevalence trend, the enrolled subjects have also been divided into groups according to their age, regardless of their vaccination status. There was no significant difference in the percentage of seronegatives (*p* > 0.05) among the groups aged 19–42 (Figure 2). On the contrary, a consistent increase of seropositives, up to almost 100%, was observed in subjects over 43 years old, therefore born before the introduction of measles vaccine in Italy. As far as seronegativity rates are concerned, some differences among the age groups are also worthy of being mentioned. All negative subjects in the 37–42 age group had never been exposed to the virus; most of the subjects aged 31–36 had been vaccinated with just one dose, while the majority of the 19–24 age group had been vaccinated with two doses. Regarding the group aged 25–30, the percentage of seronegatives was equally distributed between those vaccinated with one or two doses of vaccine.

### 3.3. Decline of Humoral Response to Measles after Vaccination

**Figure 3:**
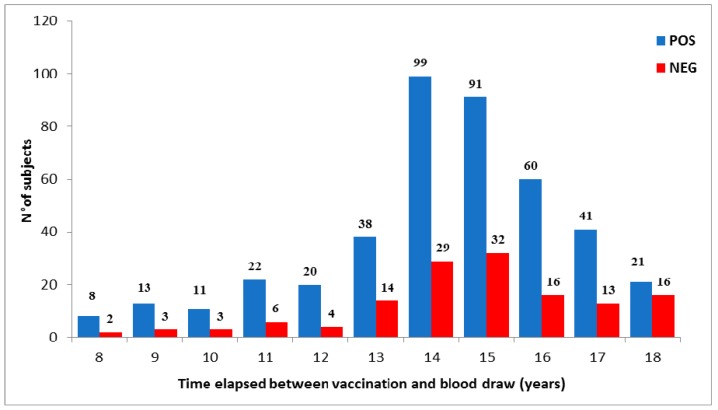
Seroprevalence of measles antibodies after vaccination over-time. Antibody response in vaccinees was analyzed 8–18 years after complete vaccination. The number of seropositive and seronegative subjects was plotted.

Figure 3 reports the seroprevalence analyzed during the period of 8–18 years after two doses of vaccination in 562 subjects within the same range of age (mean age 24.4 years; CI 95% 24.2–24.6). Although the number of tested subjects decreased over time, it was evident that an increasing number of vaccinees became seronegative in parallel with the increase of time post-vaccination. We noticed a higher percentage of seronegative subjects between 13 and 16 years after vaccination, suggesting that the decrease of the humoral response could be due to a measles-specific antibody titer after vaccination lower than after natural infection. However, this hypothesis could not be endorsed since the antibody titers after vaccine administration were not available. Extending the monitoring time, the trend was similar, although the serological profile appeared variable, probably due to the limited number of tested samples. No specific IgM were detected in any tested sample.

### 3.4. Evaluation of Neutralizing Ab Titers

**Figure 4:**
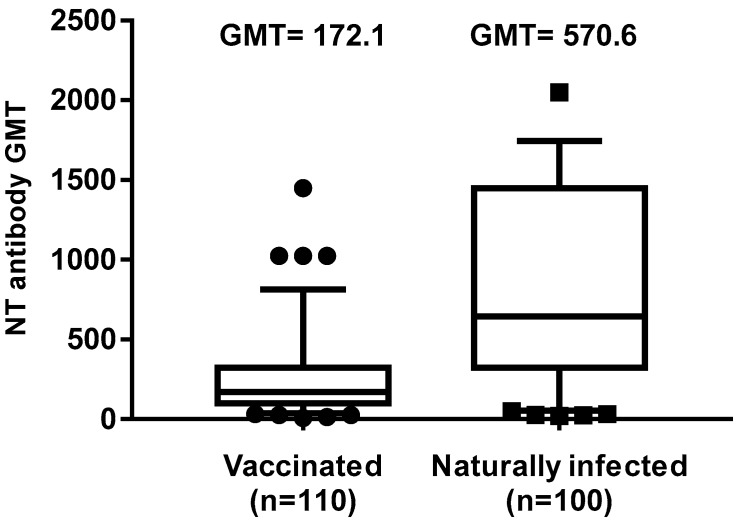
Differences in neutralizing antibody titers between subjects vaccinated with two doses of measles vaccine and naturally infected subjects are shown. The whiskers represent the values from the 5th to the 95th percentiles; the median, the 25th and 75th percentiles are depicted by the horizontal lines in the boxes. Individual data points are shown; outlier values are shown as black circles or squares. GMTs are shown above the population columns. *p*-value of the GMT between the two groups is ≤0.00001.

One hundred and ten sera of seropositive subjects who had received two doses of the measles vaccine and 100 sera of randomly selected subjects who had contracted a natural infection were tested for the presence of neutralizing antibodies against the virus. With regard to naturally infected subjects, the GMT of the tested samples was 570.6 and no substantial differences were found between males and females (*p* = 0.46). Among those vaccinated with two doses of measles vaccine, the GMT was 172.1, which was considerably lower than that recorded in naturally infected subjects (*p* < 0.00001). Moreover, no gender related significant differences in GMT were found during this analysis (*p* = 0.38) (Figure 4).

### 3.5. Decline of Neutralizing Ab Titers

**Figure 5:**
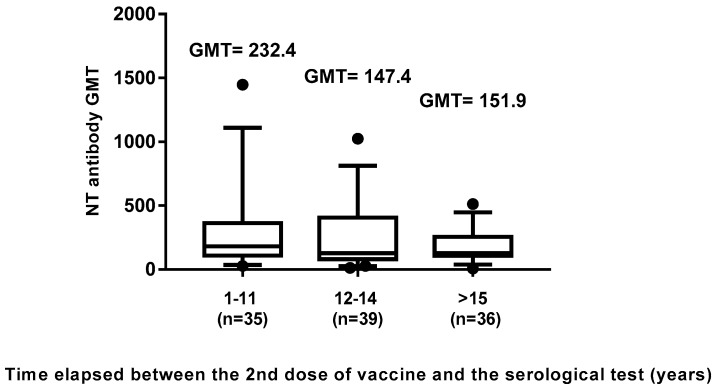
Time elapsed between the second dose of vaccine and the serological test (years). Effects of negative correlation based on the time elapsed between the second dose of measles vaccine and the last measles investigation test on neutralizing antibody response. The whiskers represent the values from the 5th to the 95th percentiles; the median, the 25th and 75th percentiles are depicted by the horizontal lines in the boxes. Individual data points are shown; outlier values are shown as black circles. GMTs are shown above the population columns. *P*-value of the GMT between the first (1–11) and second (12–14) group is <0.05.

Since previous studies reported that the measles protective antibody titer was decreasing over time after the administration of the second dose of MMR vaccine [17,18,19,20,21], our aim was to evaluate this phenomenon in our cohort. The vaccinated subjects (110) were divided into three groups, on the basis of the time elapsed since the administration of the second dose of vaccine: eleven years (35 subjects), 12–14 years (39 subjects) and ≥15 years (36 subjects). Results showed a significant decrease in GMT between the groups tested at 1–11 and 12–14 years after vaccination (232.4 vs. 147.4; *p* < 0.05) (Figure 5), thus confirming a possible decline of the protective antibody response against measles during their lifetime. No further difference was evidenced after ≥15 years since vaccination.

**Figure 6:**
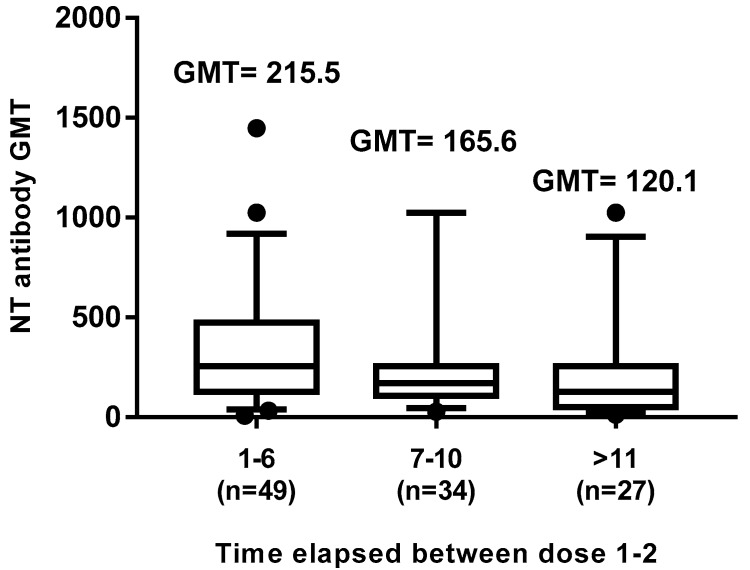
Effect of time elapsed between the two doses of measles vaccine on the neutralizing antibody response. The whiskers represent the values from the 5th to the 95th percentiles; the median, the 25th and 75th percentiles are depicted by the horizontal lines in the boxes. Individual data points are shown; outlier values are shown as black circles or squares. GMTs are shown above the population columns. The difference in GMTs between the first (1–6) and the third (≥11) group is statistically significant (*p* ≤ 0.05).

Subjects vaccinated with two doses were further studied on the basis of the time elapsed between the first and second dose of vaccine: 1–6 y (49 subjects), 7–10 y (34 subjects), and ≥11y (27 subjects). Spearman’s rank correlation analysis showed a statistically significant (*p* = 0.004) inverse correlation (r = −0.270), between neutralization titers of subject sera and time elapsed between the two-dose vaccination. Indeed, the GMT comparison among the three groups evidenced a decrease of neutralizing GMTs, 215.5 in the 1–6 y, 165.6 in the 7–10 y, and 120.1 in the ≥11 y groups (*p* < 0.05) (Figure 6), particularly relevant in subjects receiving the second dose of vaccine over 10–11 years since the first vaccination (*p* < 0.05). No significant differences related to the gender were found in this analysis either.

### 3.6. Neutralizing Antibody GMT in Natural Infected Subjects

**Figure 7:**
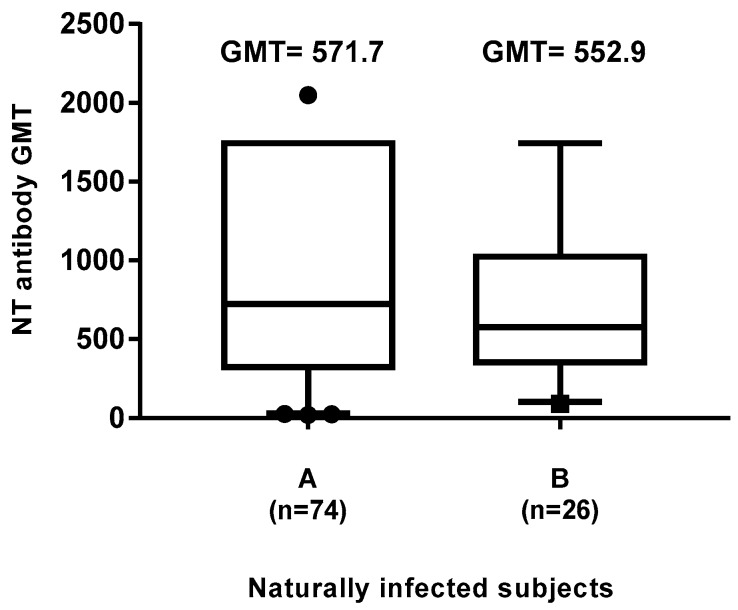
Differences in neutralizing antibody titers in naturally infected subjects born before (A) and after (B) 1977 (one year after the introduction of measles vaccine in Italy). The whiskers represent the values from the 5th to the 95th percentiles; the median, the 25th and 75th percentiles are depicted by the horizontal lines in the boxes. Individual data points are shown; outlier values are shown as black circles or squares. GMTs are shown above the population columns. Difference in GMT between the two groups is not significant (*p* > 0.05).

No GMT differences were found in serum samples of naturally infected subjects during their childhood, either in those born before (GMT 571.7; mean age 54.8) or after (GMT 552.9; mean age 31.0) the introduction of measles vaccine (Figure 7). These data confirmed a more pronounced tendency of the protective immune response to wane in vaccinated people than in naturally infected people.

## 4. Discussion

Measles morbidity and mortality have been reduced since the implementation of enhanced vaccination strategies [27], and in several countries the interruption of indigenous transmission of measles disease has been reported [28]. Unfortunately, the measles disease continues to be difficult to eradicate, especially in European countries where recurrent outbreaks have been described [29]. In Italy, although a significant reduction of measles infection has been obtained since the introduction of the measles vaccine in 1976, the goal of measles eradication fixed by WHO Europe has still not been met. A new law, adopted in Italy in July 2017 [13], extended the number of mandatory vaccines from four to 10 vaccines for those aged 0–16. This law allowed the vaccine coverage for MMR vaccine to increase up to 94.1% (range 82.2–97.5) but still with geographical variations throughout the country [30]. This program aimed at preventing widespread measles transmission [31], by inducing a specific humoral response in the community, which is a good indicator of protection from infection.

In this study, we evaluated the immunization status and the seroprevalence of measles antibodies among a healthy adult population after vaccination or natural infection. In particular, the aim of this study was to investigate the persistence of anti-measles IgG among vaccinated and naturally infected people. Collected data showed that, out of the 1092 enrolled subjects, 682 (62.45%) had received two doses of measles vaccine (monovalent and/or MMR) and among these, only 24 subjects received as first dose the monovalent vaccine, since this was replaced in the early 1990s by the combined measles–mumps–rubella (MMR). We noted that 23% of the subjects who received two doses of measles vaccine did not show IgG response, similarly to those receiving only one dose of vaccine (25%) (Figure 1). This percentage appeared higher than that reported by other authors [17,20,32,33], thus, it is advisable to monitor the immune status of vaccines 10–15 years since vaccination, in order to evaluate the immune protection against measles and, eventually, implement a possible prophylactic measure. On the other hand, a long-term high rate of seropositivity persisted after natural infection; indeed, subjects enrolled in this study who reported measles infection history were all seropositive. Therefore, we evaluated the presence of the neutralizing antibody response to the virus in naturally infected subjects and in those vaccinated with two doses. The geometric mean titer to measles was high (GMT 552.9) in naturally infected people, versus a GMT of 172.1 in vaccines of the same age cohort, indicating that the antibody titer decline was more evident in the vaccinated people, probably because the starting antibody titer after vaccination was lower than that induced after natural infection [34]. This finding is also supported by the observation that a measles virus antibody titer decrease has been revealed in source plasma donors after the introduction of measles vaccination in the United States [35], resulting in a high level of immunity and limited spread of the virus, but lower antibody level in the population. Indeed, in this study, the neutralizing antibody titer inversely correlated with the time elapsed between the second dose of vaccine and the blood drawn. This trend was confirmed (r = −0.11) by a gradual decrease of GMT titers (Figure 4), concomitant with the increase of years spent since vaccination, particularly over 12–14 years (GMT 232.4 vs. 147.4). Finally, a new interesting factor considered in this analysis was that most of the subjects had been vaccinated at different lifetimes with two doses, because of the change of the national vaccination schedule in 2003. Therefore, this parameter was analyzed to understand whether the interval of time elapsed between the two doses of vaccine could influence the immune response. It is known that schedules which have longer intervals between vaccine doses usually lead to higher immune responses [36,37,38]. On the contrary, our results, obtained by the neutralization assay on serum samples of subjects vaccinated with two doses (Figure 5), showed a different picture. An inverse correlation between neutralizing titer and time elapsed between the two vaccinations was evidenced (r = −0.27) with a significant decline of the GMT when the interval was ≥11 years in comparison to that observed within the 1–6 years interval (*p* = 0.02). The result could be very useful for the formulation of the vaccination schedule, because this finding is not valid for all vaccinations and represents a further variable factor to be considered when new vaccinations are requested. We did not find the gender factor as a variable for the immune response to measles vaccination, since no significant difference was recorded. Nowadays, measles can be easily prevented through a two-dose vaccination; however, the estimated rate of antibody decline along the time upon vaccination might represent a factor to be taken into account to limit the number of people susceptible to measles infection in the future. In this work, we only based our study on the humoral response to measles, without considering the role of cell-mediated immunity for the induction of protective immunity [39], which might be present and re-stimulated in a subject who does not apparently reveal a specific antibody response. The above discussed data suggest that there is still a small nonimmunized portion of adults, thus it is necessary to continue the monitoring of the level of protection in the population in order to limit the endemic circulation and outbreaks of measles. Finally, it is interesting to examine the immune response in depth and in its entirety and evaluate the possibility of a booster vaccination, reassessing the multiple factors that can affect and improve the immunogenicity and efficacy of the vaccine.

## 5. Conclusions

Although MMR vaccine is very efficacious to induce a protective response against measles, considering that a physiological decline of the humoral response has been revealed in some studies [35], it could be important to monitor the population 10–15 years after vaccination, in order to revaccinate the seronegatives. However, since booster revaccination might not induce a sustainable increase of MV antibodies [40], it could be advisable to implement the prophylaxis with alternative vaccines that could obviate this possibility.
